# The implication of long-lasting insecticide-treated net use in the resurgence of malaria morbidity in a Senegal malaria endemic village in 2010–2011

**DOI:** 10.1186/s13071-015-0871-9

**Published:** 2015-05-13

**Authors:** Amélé N Wotodjo, Vincent Richard, Sylvie Boyer, Souleymane Doucoure, Nafissatou Diagne, Aissatou Touré-Baldé, Adama Tall, Ngor Faye, Jean Gaudart, Jean-Francois Trape, Cheikh Sokhna

**Affiliations:** Unité de Recherche sur les Maladies Infectieuses et Tropicales Émergentes, IRD198, UM63, CNRS7278, INSERMU1095, Aix-Marseille Université, Campus UCAD-IRD, BP 1386, CP 18524 Dakar, Sénégal; Institut Pasteur de Dakar, Unité d’Epidémiologie/ Unité d’immunologie, Dakar, Sénégal; Aix-Marseille Université, UMR912 SESSTIM (INSERM-IRD-AMU), Marseille, 13005 France; Université Cheikh Anta Diop de Dakar, Faculté des Sciences et Techniques/Laboratoire de Parasitologie, Dakar, Sénégal; Institut de Recherche pour le Développement, laboratoire de Paludologie, Dakar, Sénégal

**Keywords:** Malaria, Resurgence, Long-lasting insecticide treated bed-nets, DIELMO, Senegal

## Abstract

**Background:**

Although the burden of malaria has significantly declined in recent years in sub-Saharan Africa through the widespread use of long-lasting insecticide treated bed-nets (LLINs) and artemisinin-based combination therapy, resurgence of malaria is observed in some settings after several years of LLINs use. This study aimed to assess if LLINs use remains protective against malaria during a period of resurgence of malaria morbidity in Dielmo, a rural village of Senegal.

**Methods:**

In July 2008, LLINs were offered to all villagers and lately in July 2011, LLINs were renewed.

A longitudinal study was conducted between July, 2010 and December, 2011 among inhabitants of the village of Dielmo to identify all episodes of fever. Thick smears stained with Giemsa were done for every febrile villager and malaria attacks were treated with combination of Artesunate plus Amodiaquine. Cross-sectional surveys were also conducted at the end of the rainy season (October 2010 and November 2011) to assess asymptomatic carriage. A survey on LLINs use was done every quarter of the year. A random-effect logistic regression was used to assess the effect of LLINs use on the risk of having a malaria attack after adjusting for the main risk factors.

**Results:**

The study population included 449 individuals corresponding to a total of 2140 observations. One hundred and fifteen (115) clinical malaria attacks attributed to *P. falciparum* (cases) have been recorded over the study period. Most of the malaria cases occurred in October-December 2010 (49/115 i.e. 43%) and among adults aged 15 years and over (50/115, i.e. 43%). During the study period, the use of LLINs was 61% among non-malaria cases and only 42% among malaria clinical cases but differenced according to age group.

After adjusting on gender, age, rainfall and LLINs replacement, we found that LLINs use (AOR [95%CI] = 0.40 [0.25; 0.62], p < 0.001) remained a protective factor against malaria attacks during the study period.

**Conclusion:**

LLINs use remains effective to reduce malaria burden. These results highlight the need to pursue LLINs implementation in the current context of malaria elimination and to provide positive incentives to increase its use in the population.

## Background

A subsequent decline of malaria morbidity and mortality has been achieved in recent years in sub-Saharan Africa through the widespread use of artemisinin-based combination therapy (ACT) and long-lasting insecticide-treated nets (LLINs) [1-4]. Between 2000 and 2010, nearly half of the countries affected by the disease were able to reduce the number of malaria cases by more than 50% but the intensity of malaria decrease varies according to the endemic status of the area [5].

With 207 million cases and 62,700 deaths related to malaria in the world in 2012, this disease remains a major public health concern. While the strong decline of malaria burden has encouraged the goal of pre-elimination and elimination of malaria [6,7], several concerns about the future of malaria elimination efforts are emerging. First, pyrethroid resistance of *Anopheles* has been rapidly emerging [8,9]. Second, the impregnated nets are known to act by reducing or removing human-vector contact, which could lead to a decrease of immunity in endemic areas among persons who were previously immune [9]. Third, the protective effect of LLINs use became debatable over the long term, especially in malaria endemic area. In Malawi, a recent study showed that after five years of malaria prevalence decrease thanks to the intensification of control measures, the downward trend is no longer significant [10]. Similarly, a study conducted between 2002 and 2010 after the introduction of LLINs in three sentinel sites in Kenya showed that malaria prevalence significantly decreased between 2002 and 2006 but increased in two of the three sites in 2010 [11].

In the rural village of Dielmo in Senegal, ACT and LLINs have been implemented in June 2006 and July 2008 respectively [9]. LLINs introduction has been followed-up by a sharp decrease of *P. falciparum* malaria morbidity which was found to be 13-times lower between August 2008 and August 2010 than in the period between January 2007 and July 2008 [9]. However, a resurgence of malaria morbidity has been observed between September and December 2010, i.e. 27–30 months after the nets introduction [9]. A previous study published in 2011 suggested that *Anopheles* resistance to pyrethroid could be a major factor in the increase of malaria morbidity in 2010 [9]. However, it does not explore the role played by LLINs use in the malaria upsurge period, especially whether its inhabitants had used it consistently over the period and whether LLINs remained a protective factor against malaria attacks.

In this study, we aimed to document the evolution of LLINs use over the long term and to assess its role in malaria protection during the resurgence of malaria clinical attacks using longitudinal data from the Dielmo project.

## Methods

### Setting: the Dielmo site

Since June 1990, a long-term research project is being conducted among the population of Dielmo, a Senegalese endemic malaria village, to understand the relationship between malaria incidence, its transmission, and population immunity against different *Plasmodium* species.

The Dielmo research site has been described in detail elsewhere [12]. Briefly, the village is located in a Sudan-savannah region of central Senegal at 280 km south-east of Dakar on the marshy bank of the Nema, a small permanent stream where the persistence of anopheline breeding sites is observed year-round. Malaria transmission is intense and perennial with respectively 89 and 76 infected bites per person per year in 2010 and 2011 [13]. The village comprises respectively a population of 468 and 485 inhabitants in 2010 and 2011 [13] living in 42 concessions with a median of 8 (2; 31) persons per house. Most of the inhabitants are farmers. The serer ethnic group represented 78% of the population while 13% and 9% of the villagers were respectively Mandingue and miscellaneous [12].

### Participants and procedures

All villagers of Dielmo willing to participate are involved in a longitudinal follow-up including three main components: i) daily home-based medical surveillance of all episodes of fever including treatment and prevention of malaria attacks through ACT and LLINs; ii) repeat cross-sectional surveys to document malaria prevalence; iii) quarterly repeat cross-sectional surveys to document LLIN use. Written informed consent was obtained from all participants or from the guardians or the parents of children enrolled. The study was approved by the Ministry of Health of Senegal, the assembled village population and the National Ethics Committee of Senegal.

#### Home-based medical surveillance

All participating households were registered with key socio-demographic information (household composition and age, gender of its different members) and visited daily. The presence or the absence in the village of each enrolled household members was monitored and location of absent member was reported. This enables the identification of a resident member (defined by spending at least 75% of time during the trimester in Dielmo) and to compute the number of followed up person-days under observation for each period. Body temperature was systematically recorded at home three times a week in children younger than 5 years, and in older children and adults in cases of suspected fever or fever-related symptoms. In case of fever, patients were referred to the project health centre which was open 24 h a day, 7 days a week and examined by a nurse. Thick smears stained with Giemsa were performed and the presence of parasite on thick smears was determined using light microscopy. The parasite leukocyte ratio was measured for each plasmodial species. Episodes of fever were attributable to *P. falciparum* malaria clinical attacks when parasite density was higher than an age-dependent threshold [14]. The threshold for *P. falciparum* attacks was ranged from a maximum of 125 trophozoites per 100 leucocytes in children aged 2 years to a minimum of 6 trophozoites per 100 leucocytes in adults aged 60 years and over [14]. Malaria clinical attacks were treated with combination Artesunate plus Amodiaquine by June 2006 until now. The efficacy of the treatment was monitored by daily clinical surveillance of patients and with at least one control of parasitaemia between day 7 and day 35 after fever resolved. In addition, the type of hemoglobin, the ABO and rhesus blood group were systematically performed for all enrolled individuals at the inclusion in the project and consigned in a biological data bank [12].

#### Cross-sectional surveys

To assess asymptomatic carriage and malaria prevalence, cross-sectional surveys were conducted at the end of the rainy season (in October 2010 and November 2011) when mosquitoes were abundant and when the transmission was very high. All individuals enrolled in the Dielmo project that were present in the village at the time of the survey were eligible to participate.

#### Quarterly LLINs repeat cross-sectional surveys

LLINs (Permanet 2.0) were introduced for the first time in the village in July 2008, where they have been offered to all villagers. Three years after, in July 2011, all LLINs were renewed. Simultaneously to the introduction of LLINs, repeat home-based surveys have been carried out to assess their use. Each participating household was visited quarterly in the morning by two technicians in charge of recording whether the nets were hung above the bed the night before and of administrating to household members a short questionnaire about LLINs use. Individuals were asked if they had used nets the night preceding the visit and whether they never, always or sometimes used nets.

All collected data were entered into the 4D software version 2004.5.

### Study population

In our study, we focused on clinical observations covering the period from July 2010 to December 2011 where rebound of malaria incidence has been observed. All inhabitants of Dielmo who were enrolled in the project during this period and for whom data on LLINs use were available (i.e. participants who were investigated for LLINs use) were included in the study. Exclusion criteria included pregnant women which represent a group with specific malaria risk factors.

### Outcome definition

Malaria clinical attacks occurring among our study population were gathered together in 6 quarters including July-September 2010 (period 1); October-December 2010 (period 2); January-March 2011 (period 3); April-June 2011 (period 4); July-September 2011 (period 5) and October-December 2011 (period 6). Our analysis was thus based on person-trimester observations. Malaria cases were defined during the quarter as individuals who had at least one malaria clinical attack. For each trimester, we defined two groups according to the presence or absence of malaria clinical attacks (malaria cases were coded 1 and non- malaria cases were coded 0).

### Statistical analysis

We calculated incidence rates of clinical malaria attacks as the ratio of the number of clinical malaria attacks recorded during a given period divided by the number followed up person-days under survey during the corresponding period. We derived mean monthly incidences rate by period from the daily incidence rates on the basis of 30.4 days per month.

Factors associated with the outcome variable were investigated using a random-effect logistic regression model, which took into account the interdependence of successive observations for the same individuals (as we used person-trimester observations) [15].

The following socio-demographic and biological variables were investigated in the analysis: sex, age group (defined in 6 groups according to the literature [9,12]), education (went to French school or not), being resident during the quarter, type of hemoglobin, ABO group and rhesus group. We also included in the analysis information on rainfall defined by the cumulative number of mm of rainfall during the previous quarter in order to take into account the time gap between the occurrence of rainfall and the recrudescence of malaria attacks. Finally, information on LLINs included two variables: LLINs use and LLINs replacement. LLINs use was introduced using a dichotomous variable: “always use LLINs” versus “not consistently use LLINs”; this second category grouped together individuals who reported to “sometimes” or “never” use LLINs during the corresponding quarter as well as individuals who do not have LLINs. LLINs replacement was also defined as a dichotomous variable indicating the time at which the nets have been renewed (i.e. before and after July 2011).

First, univariate analyses were performed to identify significant association. Factors with a P-value below 0.20 in univariate analyses were considered eligible for the multivariate analysis. Second, a stepwise backwards-elimination approach based on the Akaike criteria was used to select the variables to keep in the final model. In addition, the final model was adjusted for age groups to control for demographic characteristics. The significance level was fixed at p < 0.05 in the final model.

Analysis were performed using Stata Software version 11.0 (College Station, Texas, USA) and R software.

## Results

### Description of participants

**Figure 1:**
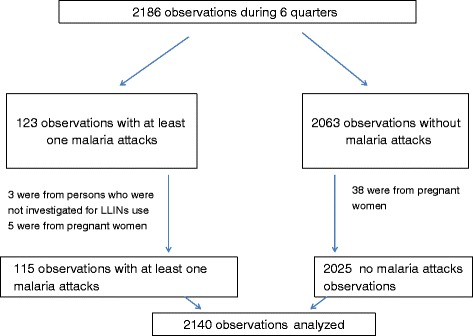
Study observations profile.

We observed 2186 observations corresponding to 452 individuals during the study period. Among the 2186 observations, we excluded 3 observations among malaria cases because they were not investigated for LLINs surveys and another 43 which were related to pregnant women. We finally obtained 2140 observations corresponding to 449 individuals aged from one month to 100.7 years old with a mean of 23.4 years old and a proportion of 49% of women. Among the 2140 observations, 115 (5.6%) were related to individuals who had at least one malaria attack per trimester between July 2010 and December 2011 and 2025 observations were related to individuals who had no malaria attacks. The profile of the study population was shown in Figure 1.

### Incidence of malaria clinical attacks over the study period

**Figure 2:**
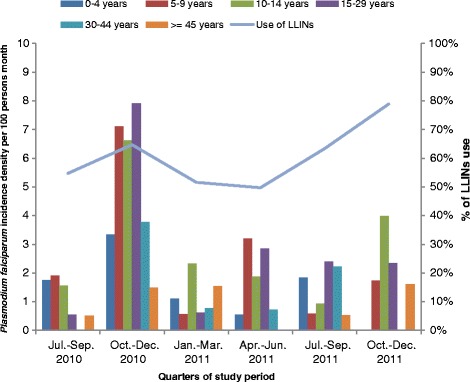
Monthly incidence of *Plasmodium falciparum* by age group and use of LLINs according to the periods of study.

Two hundred and ninety seven (297) and 292 thick smears were performed in October 2010 and November 2011 respectively during cross-sectionnal surveys. *P. falciparum* prevalence was 2.7% (8/297) in 2010 and 1.4% (4/292) in 2011. Table 1 describes the number of malaria clinical attacks and monthly incidence by age-groups and by periods while Figure 2 illustrates both the time trends of monthly incidence (by age-groups and by periods) and of LLINs use. Among the 115 malaria clinical cases, 10 to 16 cases were recorded at each time period, except during the second period (October-December 2010) where 49 of the malaria clinical attacks were observed.

**Table 1:** **Number of follow-up days and** ***Plasmodium falciparum*** **morbidity by age group according to the periods of the study**

	**0-4 years**	**5-9 years**	**10-14 years**	**15-29 years**	**30-44 years**	**> = 45 years**	**Total**
July-September 2010							
Follow-up days	5218	4766	3897	5488	3553	5938	28860
Malaria attacks	3	3	2	1	0	1	10
Monthly incidence per 100 persons	1.748	1.914	1.560	0.554	0.000	0.512	1.053
October-December 2010							
Follow-up days	5444	4701	4587	5384	4023	6113	30252
Malaria attacks	6	11	10	14	5	3	49
Monthly incidence per 100 persons	3.350	7.113	6.627	7.905	3.778	1.492	4.924
January-March 2011							
Follow-up days	5475	5289	5206	4971	3902	5911	30754
Malaria attacks	2	1	4	1	1	3	12
Monthly incidence per 100 persons	1.111	0.575	2.336	0.612	0.779	1.543	1.186
April-June 2011							
Follow-up days	5533	5695	4863	5319	4219	5689	31318
Malaria attacks	1	6	3	5	1	0	16
Monthly incidence per 100 persons	0.549	3.203	1.875	2.858	0.721	0.000	1.553
July-September 2011							
Follow-up days	4972	5113	3281	5069	4088	5703	28226
Malaria attacks	3	1	1	4	3	1	13
Monthly incidence per 100 persons	1.834	0.595	0.927	2.399	2.231	0.533	1.400
October-December 2011							
Follow-up days	4985	5257	3812	5174	3992	5642	28862
Malaria attacks	0	3	5	4	0	3	15
Monthly incidence per 100 persons	0	1.735	3.987	2.350	0	1.616	1.580
All the study period							
Follow-up days	31627	30821	25646	31405	23777	34996	178272
Malaria attacks	15	25	25	29	10	11	115
Monthly incidence per 100 persons	1.442	2.466	2.963	2.807	1.279	0.956	1.961

The incidence density of *P. falciparum* malaria attacks was thus more than 3 fold higher in October-December 2010 with 4.92 attacks per 100 persons per month compared with 1.05 in July-September 2010 (the lowest incidence) and 1.58 in October-December 2011 (the second highest incidence over the period). Over the whole study period, the incidence density was also the highest among the age groups 10–14 years old, 15–29 years and 5–9 years with respectively 2.96, 2.81 and 2.47 attacks per 100 persons per month versus 1.44, 1.28 and 0.96 in age groups < 5 years, 30–44 and >45 years, respectively. The three age groups 10–14 years old, 15–29 years and 5–9 years were also those with the highest incidence density during the October-December 2010 period (incidence density = 6.63, 7.91 and 7.11 per 100 persons per month, respectively).

### Description of LLINs use during the study period

**Figure 3:**
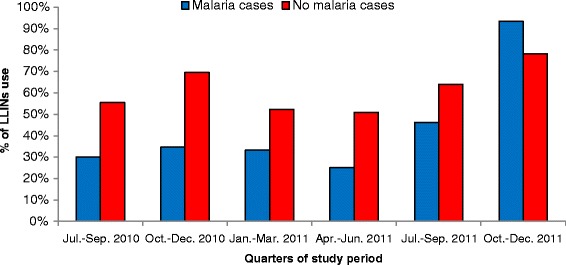
% of LLINs use according to the absence or presence of malaria at the different time periods.

**Figure 4:**
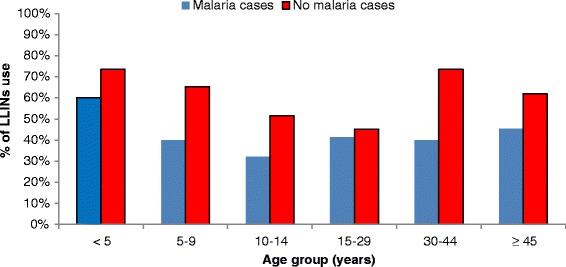
% of LLINs use by age groups according to the absence or presence of malaria.

Figure 3 shows the proportion of LLINs use according to the presence or absence of malaria attacks and according to the study period and Figure 4 illustrates the proportion of LLINs use according to age groups and the presence or absence of malaria attacks.

We observed that the use of LLINs was quite stable over the study period with 197/360 (55%), 231/357 (65%), 191/370 (52%), 181/364 (50%), 223/352 (63%) and 266/337 (79%) observations corresponding to individuals who always used LLINs at each of the different time periods. The proportion of LLINs use was also systematically lower among the group of malaria cases than among the group without malaria cases for each of the periods, except for the last one (October-December 2011) where the proportion of LLINs use was 93% (i.e. 14/15) among malaria cases versus 78% (i.e. 252/322) among the group without malaria cases.

Overall, the proportion of LLINs use was the highest among children aged less than 5 years (268/367 i.e. 73%) and the lowest among the 10–14 years and 15–29 years age group (152/304 i.e. 50% and 194/432 i.e. 45% respectively) (Figure 4). For all age groups, the proportion of LLINs use was lower than 50% in malaria cases except in children less than 5 years for whom the proportion of nets used was about 60% (9/15) in malaria cases. In addition, we observed that for all age groups the proportion of LLINs use was systematically lower among the group of malaria cases except in the 15–29 years age groups where the proportion of LLINs use was approximately similar in the two groups (12/29 i.e. 41% in the malaria group versus 182/403 i.e. 45% in the non- malaria group).

### Factors associated with the risk of having malaria clinical attacks

Tables 2 and 3 respectively describe individual characteristics for both the whole study population and according to the two groups previously defined (i.e. observations with malaria attacks and observations without malaria attacks) and results of univariate and multivariate analysis of the risk of having malaria attacks. We observed that the proportion of women was lower in the group with malaria attacks than in the group without malaria (43/115 i.e. 37% versus 1,013/2,025 i.e. 50%). With an OR [95% CI] of 0.59 [0.39; 0.90], female gender was found to be a protective factor in univariate analysis. As already shown with the results on incidence density, the proportions of individuals aged 5–9 years, 10–14 and 15–29 were higher in the group with malaria than in the group without malaria. In univariate analysis, however, the age group 10–14 years was the only age group significantly associated with a higher risk of malaria compared with children aged less than 5 years (OR [95%CI] =2.07 [1.03; 4.17]). Almost all cases (100/115 i.e. 87%) were found in resident individuals but the proportion of resident in the group without malaria attacks was similar (1787/2025 (88%)) and being resident was not found to be associated with the risk of malaria in univariate analysis (OR [95%CI] =0.89 [0.49; 1.59]). Biological characteristics were also quite similar in the two groups and none significant association was found in univariate analysis. The type of hemoglobin AA was the most represented with 1572/2140 (73.5%) observations in the whole study population while only 143/2140 (6.7%) observations were from the AS type and 11/2140 (0.5%) from the AC type. The A and O blood groups represented respectively 550/2140 (25.7%) and 743/2140 (34.7%) of the whole study population. Finally, the proportion of observations recorded in individuals who always used LLINs (1241/2025 i.e. 61.3%) was higher in the group without malaria than in the group with malaria (48/115 i.e. 41.7%). In univariate analysis, the use of LLINs was found to be a protective factor of malaria clinical attacks during the study period (OR [95%CI] = 0.45 [0.30; 0.68], p < 0.001). While the replacement of LLINs in July 2011 tend to be a protective factor of the risk of having malaria attacks, the association was not significant at the 5% threshold in univariate analysis (OR [95%CI] =0.65 [0.42; 1.02], p = 0.06). Finally, rainfall was significantly associated with a higher risk of malaria (OR [95%CI] =1.0017 [1.001; 1.002], p < 0.001).

**Table 2:** **Socio-demographic and biological characteristics among the whole population and according to the absence or presence of malaria attacks (n = 2,140)**

**Characteristics**	**Subcategory**	**Number of persons n = 449**	**Number of observations n = 2,140 n(%)**	**Malaria cases**	
				No n = 2025 n(%)	Yes n = 115 n(%)
Socio-demographic characteristics					
Sex					
	Male	-	1084 (51)	1012(50)	72 (63)
	Female	-	1056 (49)	1013 (50)	43 (37)
Resident					
	Yes	-	1887 (88)	1787 (88)	100 (87)
	No	-	253 (12)	238 (12)	15 (13)
French School					
	Yes	37 (8)	155 (7)	144 (7)	11 (10)
	No	362 (81)	1764 (82)	1672 (83)	92 (80)
Age group					
	<5 years old	-	367 (17)	352 (17)	15 (13)
	5-9 years old	-	353 (17)	328 (16)	25 (22)
	10-14 years old	-	304 (14)	279 (14)	25 (22)
	15-29 years old	-	432 (20)	403 (20)	29 (25)
	30-44 years old	-	282 (13)	272 (13)	10 (9)
	45 years old and over	-	402 (19)	391 (19)	11 (10)
Periods					
	Jul.-Sep. 2010	360 (17)	360 (17)	350 (17)	10 (9)
	Oct.-Dec. 2010	357 (17)	357 (17)	308 (15)	49 (43)
	Jan.-Mar. 2011	370 (17)	370 (17)	358 (18)	12 (10)
	Apr.-Jun. 2011	364 (17)	364 (17)	348 (17)	16 (14)
	Jul.-Sep. 2011	352 (17)	352 (17)	339 (17)	13 (11)
	Oct.-Dec. 2011	337 (16)	337 (16)	322 (16)	15 (13)
Biological characteristics					
ABO Group					
	A	116 (26)	550 (26)	514 (25)	36 (31)
	B	78 (17)	354 (17)	331(16)	23 (20)
	AB	16 (4)	80 (4)	77 (4)	3 (3)
	O	148 (33)	743 (35)	703 (35)	40 (35)
HB type					
	AA	325 (72)	1572 (74)	1479 (73)	93 (81)
	AS	30 (7)	143 (7)	8 (7)	8 (7)
	AC	3 (1)	11 (1)	11 (1)	0 (0)
Rhesus group					
	+	355 (75)	1617 (76)	1518 (75)	99 (86)
	-	23 (5)	110 (5)	107 (5)	3 (3)
Nets use characteristics					
LLINs Use					
	Always		1289 (60)	1241 (61)	48 (42)
	Not always		767 (36)	707 (35)	60 (52)
LLINs replacement					
	Yes		689 (32)	661 (33)	28 (24)
	No		1451 (68)	1364 (67)	87 (76)

**Table 3:** **Random-effect logistic regression models exploring factors associated with malaria clinical cases (n = 2,140)**

		**Univariate analysis**		**Multivariate analysis**	
**Characteristics**	**Subcategory**	**OR (95% CI)**	***P*** **-value**	**AOR (95% CI)**	***P*** **-value**
Socio-demographic characteristics					
Sex					
	Male (ref)	1		1	
	Female	0.59 (0.39-0.90)	0.014	0.63 (0.41-0.98)	0.039
Resident					
	No (ref)	1			
	Yes	0.89 (0.49-1.59)	0.689		
French School	No (ref)				
	Yes	1.40 (0.67-2.89)	0.371		
Age group					
	< 5 years old (ref)	1			
	5-9 years old	1.78 (0.89-3.57)	0.103	1.77 (0.86-3.65)	0.123
	10-14 years old	2.07 (1.03-4.17)	0.041	1.82 (0.87-3.81)	0.111
	15-29 years old	1.70 (0.86-3.33)	0.124	1.16 (0.56-2.41)	0.693
	30-44 years old	0.86 (0.37-2.02)	0.731	0.81 (0.33-2.00)	0.648
	45 years old and over	0.65 (0.29-1.49)	0.310	0.59 (0.25-1.42)	0.238
Periods					
	Jul.-Sep. 2010 (ref)	1			
	Oct.-Dec. 2010	6.25 (3.03-12.87)	<0.001		
	Jan.-Mar. 2011	1.18 (0.49-2.80)	0.715		
	Apr.-Jun. 2011	1.62 (0.71-3.68)	0.248		
	Jul.-Sep. 2011	1.35 (0.57-3.16)	0.494		
	Oct.-Dec. 2011	1.65 (0.72-3.80)	0.235		
ABO Group					
	A (ref)	1			
	B	0.99 (0.56-1.76)	0.986		
	AB	0.54 (0.15-1.89)	0.336		
	O	0.81 (0.49-1.32)	0.390		
HB type					
	AA (ref)	1			
	AS + AC	0.87 (0.40-1.90)	0.730		
Rhesus group					
	+ (ref)	1			
	-	0.43 (0.13-1.42)	0.166		
LLINs use characteristics					
LLINs Use					
	Not always (ref)	1			
	Always	0.45 (0.30-0.68)	<0.001	0.40 (0.25-0.62)	<0.001
LLINs replacement					
	No (ref)	1			
	Yes	0.65 (0.42-1.02)	0.060	0.57 (0.35-0.92)	0.021
Rainfall					
		1.0017 (1.0011-1.0023)	<0.001	1.0023 (1.0016-1.0029)	<0.001

OR: Odds ratio; AOR: adjusted odds ratio.

Most of those results were confirmed in the final multivariate model. After adjustment on age groups and rainfall, female gender (AOR [95%CI] =0.63 [0.41; 0.98], p = 0.039), replacement of LLINs (AOR [95%CI] =0.57 [0.35; 0.92], p = 0.021) and LLINs use (AOR [95% CI] = 0.40 [0.25; 0.62], p < 0.001) were independent protective factors against the risk of malaria clinical attacks.

## Discussion

Thanks to the intensification of malaria control measures over the ten last years, malaria declined in many African countries [1-4,11]. In Dielmo, a rural village of Senegal, a similar trend was observed after the implementation of LLINs and ACT as first line drug anti-malaria [9]. However, 27–30 months after LLINs introduction, malaria morbidity increased, especially in older children and adults. A previous publication highlights that this upsurge coincided with important pyrethroid resistance of *An. gambiae* [9]. The aim of this study was to explore the specific role of LLINs use on malaria resurgence that occurred in Dielmo in late 2010 and 2011. Our results showed that the use of LLINS remains a protective factor against malaria attacks despite the resurgence of malaria morbidity: individuals who had malaria were indeed less likely to use consistently their nets compared to individuals who did not have malaria. We also observed a relatively high level of LLINs use which remains quite stable during the study period (between 50% and 79%) but a lower use of nets in old children aged 10–14 years and younger adults aged 15–29 years compared with other age groups. Regarding vectors, most of the bites were still after midnight [9] and they did not become early biters during our study period. As well as in Dielmo, resurgence of malaria burden has been reported in some countries of sub-Saharan Africa [11,16,17], especially in Kenya where the lack of proper net use has been suspected to be one of the factors contributing to malaria prevalence rebound [11]. However, this study is one of the first to assess the relationships between the evolution of both LLINs use and malaria burden.

Our study results highlight the highest incidence of malaria among adolescents aged 10–14 years and young adults aged 15–29 years. Indeed, about 47% of clinical attacks occurred among these age groups during our study period compared with 22% before net implementation in 2006 and 2007 (Data not shown). The lower use of LLINs among these age groups could explain the highest incidence we observed. This could also be explained by the decrease of immunity against *Plasmodium falciparum* due to the use of LLINs. Indeed, it has been shown that living in endemic regions and being exposed continuously to mosquito bites provided immunity against clinical malaria which decreased when exposure to malaria is discontinued [18]. As impregnated nets act by reducing or removing human-vector contact, the immunity can disappear or decrease when LLINs are often used. Studies in Kenya showed that the mean age of people with clinical attacks increased steadily as exposure to mosquito declined [19].

In our study, male gender was at higher risk of malaria than female. Male gender has been found as a risk factor of malaria in some studies but the association remains inconsistent [20,21]. In Dielmo, males, especially teenagers, often slept late and strolled around the village (personal observation). Therefore, they are more vulnerable to mosquito bites than others because mosquitoes are both exophilic and endophilic in Dielmo.

Among factors independently associated with a lower risk of malaria attacks, we found that both consistent LLINs use and replacement of LLINs in 2011 had a protective effect against malaria. These results suggest that despite an increase in the frequency of pyrethroid resistance in *An. gambiae* in Dielmo (7% in 2007 to 48% in 2010 [9]), nets remain effective against malaria as they can ensure at least physical protection. Indeed, 2 meta-analysis studies have shown that impregnated nets decreased malaria incidence of about 24% or 39% in areas with stable malaria, compared to untreated nets and 50% compared with no nets [5,22]. In addition, it has been demonstrated that 50% of the protection of LLINs is due to physical aspect and 50% to the presence of insecticide [5]. Another study in Benin showed that sleeping under LLINs in an area of pyrethroid resistance provided the same protection as sleeping under untreated nets, regardless of its physical condition [23]. Some studies showed no association between the increase of pyrethroid resistance and resurgence of malaria burden or no reduction of the LLINs protective effect in the area of pyrethroid resistance [24,25]. According to these observations and to our results, LLINs remains effective against malaria burden because of its physical protection at least and remains one of the best tools to control malaria burden despite the emergence of *Anopheles* resistance to pyrethroid. In addition, a recent study suggests that to be effective, the nets dissemination campaign should include motivation for using nets [26]. The next challenge will be the communication and awareness campaign about LLINs role and about the danger to neglect it over the long term especially in a context of immunity decrease.

Our results also showed that malaria prevalence decreased since the implementation of LLINs, suggesting a decrease in infected mosquitoes and mosquito biting [9]. This decrease is also due to the use of ACT in first line treatment against clinical malaria as some artemisinin derivatives were known to have an impact on *P. falciparum* gametocytes [27-29]. To maintain the recent good outcomes against malaria, it is thus important to ensure safety and affordable anti-malaria treatment to all malaria endemic countries and take into account the vulnerability of older children and adults against malaria.

This study has some limitations. First, we could not confirm the possible presence of a decrease in the immunity against *P. falciparum* as no information on immunity was collected. However, a recent study mentioned an important decrease of seropositivity against crude extracts of *P. falciparum* schizonts among Dielmo population between 2000 and 2010 [30]. Second, we observed a slightly higher proportion of LLINs use in the group of malaria clinical cases compared with the non- malaria cases group in the last period (October-December 2011). The difference between the two groups was however not significant.

We had controlled education status and there is no association between education and malaria risk. In Dielmo, almost all villagers are farmers and nets were distributed freely to all inhabitants, so the socio-economic status could not reflect residual confounding. Even if the results were adjusted on the main known factors, some confounders could be missed. We are convinced that these missed minor factors would not greatly change the results.

## Conclusion

This study showed that LLINs remain effective despite the resurgence of malaria. This resurgence was associated by the non-use of LLINs. Malaria morbidity in adults in this period had doubled as all new infected individuals developed symptoms of malaria, probably due to the loss of immunity. Immunological tests were needed to confirm the loss of immunity hypothesis, already highlighted by Fode *et al.* [30]. Our finding however suggest that scaling up nets among populations remains an important preventive tool against malaria morbidity, and that awareness campaign and monitoring of its use are crucial to avoid malaria resurgence.
